# Storage duration of packed red blood cells transfused during veno-venous extracorporeal membrane oxygenation is associated with elevated pulmonary artery pressure and lung injury in a sheep model

**DOI:** 10.1186/s13054-025-05438-z

**Published:** 2025-06-13

**Authors:** Fergal T. Temple, Gabriela Simonova, Margaret R. Passmore, Samuel R. Foley, Sara D. Diab, Kimble R. Dunster, Charles I. McDonald, Kiran Shekar, Yoke-Lin Fung, John-Paul Tung, John F. Fraser

**Affiliations:** 1 Research and Development, Australian Red Cross Lifeblood, Brisbane, Australia; 2 Sydney Medical School, University of Sydney, Sydney, Australia; 3 Critical Care Research Group, University of Queensland and The Prince Charles Hospital, Brisbane, Australia; 4 School of Health, University of Sunshine Coast, Sippy Downs, Australia; 5 Queensland University of Technology, Brisbane, Australia; 6 Faculty of Medicine, University of Queensland, Brisbane, Australia; 7 Queensland Children’s Hospital, Brisbane, Australia

**Keywords:** Extracorporeal membrane oxygenation, Transfusion, Red blood cell storage, Lung injury, Pulmonary artery pressure

## Abstract

**Background:**

Veno-venous extracorporeal membrane oxygenation (VV-ECMO) is associated with a high transfusion burden. While trials have concluded that red blood cell (RBC) storage does not impact patient morbidity and mortality in the critically ill or cardiac surgical cohorts, evidence is sparse for ECMO cohorts. A sheep model was to investigate this question. On an underlying injury of smoke inhalation, we compared fresh (< 5 days) or stored (35–42 days) RBC transfusion with no transfusion. Clinically relevant outcomes included pulmonary artery pressure as well as biochemical or histopathological markers of lung, liver, and renal injury.

**Methods:**

Twenty-four female Merino/Samm Border Leicester Cross sheep were anaesthetised and placed on pressure-controlled ventilation. They were instrumented for continuous haemodynamic monitoring. They then underwent smoke inhalation injury, followed two hours later by veno-venous ECMO. Sheep were randomised to three groups: control (no transfusion (*n* = 8)), fresh RBC (*n* = 8) and stored RBC (*n* = 8) transfusion occurring six hours after ECMO initiation. Blood samples were taken regularly, and 24 h post-ECMO, animals were sacrificed. Post-mortem samples of lung and kidney were collected for post-mortem analyses.

**Results:**

Following ECMO initiation and transfusion, pulmonary artery pressure increased in the stored RBC group. Histopathological analysis also demonstrated a significantly elevated lung injury in this group. This lung injury was characterised by greater extravasation of inflammatory cells as well as bronchiole damage and oedema. Renal histopathology showed no significant differences between groups. Alanine aminotransferase, aspartate aminotransferase and bilirubin levels increased in a time dependent manner post-transfusion but there were no treatment-associated differences. During experimentation, the coagulation profile changed with firmer clots forming more quickly. Differences were observed between both transfusion groups and controls.

**Conclusions:**

Transfusion of stored RBC elevated pulmonary artery pressure when compared to fresh RBC transfusion and controls. This change was correlated with a greater post-transfusion lung injury in the stored RBC group. Further investigation assessing whether insufficient nitric oxide had a role in these findings is warranted, as is consideration of longer ECMO durations and greater transfusion volumes.

**Graphical abstract:**

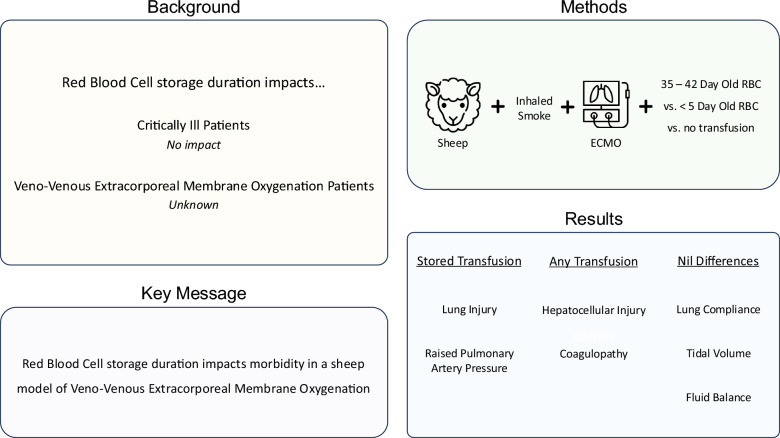

## Background

Veno-venous (VV) extracorporeal membrane oxygenation (ECMO) is increasingly being utilised as a rescue therapy for acute respiratory hypoxemia that is refractory to conventional treatment [1]. Between 2012 and 2022, the international Extracorporeal Life Support Organisation registry reported more than a tripling of annual cases with over 18,000 being completed worldwide in 2022 [2]. While VV ECMO is associated with increased survival rates in patients with acute respiratory distress syndrome [3], it is not without complications. A systematic review of 12,860 patients showed that cannulation-related complications occurred in 6.6% of VV ECMO cases; with sepsis (26%), acute renal failure (25%) and multi-organ failure (25%) substantially more likely [4]. Endothelial dysregulation is a feature of these conditions [5, 6] and there is evidence that VV ECMO can induce significant cell-junction disruption [7].

There are mechanical and chemical interactions that are non-physiological despite advancements in the biocompatibility of the ECMO ex-vivo circuitry [8]. As a result, ECMO activates inflammation [9] and the coagulation cascade [10]. These disturbances result in inappropriate clot formation and a heightened risk of bleeding [11]. Clinically, this manifests as coagulopathy in ECMO patients [11] and is associated with a major haemorrhage rate of 19% [3].

Consequently, blood products such as fresh frozen plasma, platelet concentrates, and red blood cell (RBCs) are essential in managing VV ECMO patients [12]. A meta-analysis of 3,808 patients found that VV ECMO patients were transfused an average of 19.3 RBC units per ECMO run [13]. Blood transfusion is not without risks [14] and restrictive transfusion thresholds (haemoglobin below 7 g/dL) have been shown to be as effective as liberal thresholds (< 10 g/dL) in the critically ill patient with anaemia or acute coronary syndrome [15]. A ‘restrictive’ 7 g/L haemoglobin transfusion threshold—combined with lower dose anti-coagulation—reduced bleeding events in an ECMO-only cohort (89% veno-venous configuration) [16]. When examining the possibility of a dose dependent relationship, mortality was independently associated with total blood components transfused [17].

Depending on the jurisdiction, RBCs are stored for up to 42 days [18]. During this period several biochemical and morphological changes occur, that are collectively referred to as the red cell storage lesion [19]. These changes include release of haemoglobin in both its free [20] and extracellular vesicle bound [21] forms. This possibly explains the impact of stored RBC transfusion on nitric oxide scavenging and pulmonary hypertension [21–23]. Extracellular vesicles have additionally been implicated in microthrombi formation [24].

Studies encompassing the critically ill and cardiac surgery cohorts have shown that RBC storage duration is generally not associated with adverse clinical outcomes [25, 26]. However, the relevance of these findings to less frequent interventions such as ECMO is uncertain, especially in consideration of the high transfusion requirements of these critically ill patients. Furthermore, as the RBC transfused in these studies were of a mean storage between 22 and 28 days [25, 26], the extremes of storage (i.e., 35–42 days) remain under-investigated. Laboratory evidence suggests that RBC storage lesion development accelerates from around day 28 [19] and transfusion of > 30-day old RBC units was associated with increased mortality in a meta-analysis [14]. Studying ECMO in clinical trials of large and controlled population sizes is practically very difficult [27]. One paediatric study assessed oxygenation post-transfusion via venous sampling of the ECMO circuit and near-infrared spectroscopy [28]. They found no differences betwee* n* > 21-day stored RBC and < 7 day stored blood. This study, however, was assessing veno-arterial ECMO and did not address the extremes of RBC storage. Given the practical difficulties and limited research, animal models can be a useful tool to investigate the impact of transfusing stored RBC in ECMO.

Our group has previously established a clinically relevant sheep model of RBC transfusion [29] and VV ECMO [30], and has investigated the storage lesion of sheep RBCs [31]. Previous work has shown that acute lung injury induced by smoke inhalation (s-ALI) and VV ECMO—together and in isolation—can provide insults that alter coagulation and inflammation [32, 33]. The present study used a sheep model of s-ALI followed by VV ECMO to investigate the effects of transfusing either fresh (< 5 days) or stored (35–42 days) sheep RBC, monitoring clinically significant adverse events, such as respiratory distress, inflammation, coagulopathy, and organ injury.

## Methods

This study was approved by the animal research and ethics committees of the Queensland University of Technology and the University of Queensland (approval numbers 1100000053 and 1000000025 respectively). Work adhered to the Australian Code for the Care and Use of Animals for Scientific Purposes, Eighth Edition, 2013 of the National Health and Medical Research Council.

### Transfusion preparation

Whole blood was collected from donor sheep, processed into RBC, and cross-matched as previously described [29, 31]. In brief, whole blood was collected from the right internal jugular vein. After centrifugation and separation, the red blood cells were re-suspended in additive solution (saline, adenine, glucose, and mannitol—SAGM), and each RBC unit was leukofiltered. RBC were stored at 2–6 °C for either < 5 days (fresh RBC) or 35–42 days (stored RBC).

### Study protocol

Female Merino/Samm Border Leicester Cross sheep (*n* = 24) were randomised to three treatment arms. All sheep received a smoke inhalation insult, were placed on VV-ECMO, and received protocolised clinical management as detailed below. The transfusion groups received either fresh RBC or stored RBC (each *n* = 8), with non-transfused sheep (*n* = 8) as a control group.

#### Instrumentation and continuous monitoring

**Fig. 1:**
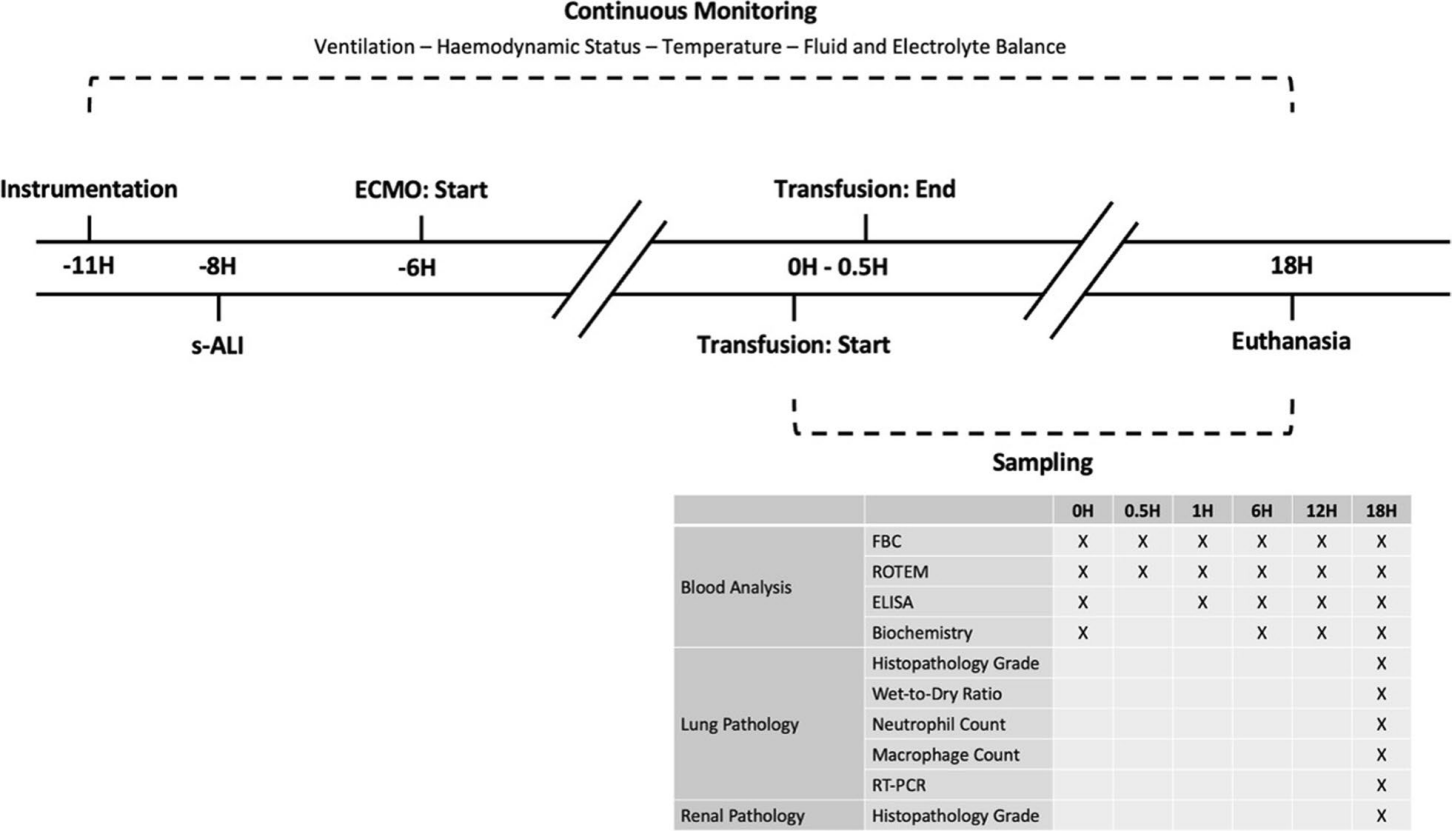
Experimental Intervention and Outcome Timeline. Experimental timeline with measurements and samples taken. FBC = full blood count, ROTEM = rotational thromboelastography, ELISA = enzyme linked immunoassay, RT-PCR = real-time polymerase chain reaction, s-ALI = smoke-induced acute lung injury

Operating theatre set-up was as previously described [34]. Under local anaesthesia, a multi-lumen central venous catheter and 8-French sheath were placed in the left internal jugular vein. Sedation was induced with midazolam (0.5 mg/kg iv; Pfizer, NSW, Australia) and alfaxalone (3 mg/kg iv; Jurox, NSW Australia). Sheep were intubated with an orotracheal tube and pressure controlled mandatory ventilation—based on positive end expiratory pressure (PEEP) and peak airway pressure—was used to protect against barotrauma [35, 36]. The internal jugular vein had three venous sheaths introduced for intracardiac echocardiography and to guide subsequent ECMO cannulation post-smoke inhalation. The facial artery was cannulated for arterial blood gas sampling and pressure monitoring. Sheep were continuously monitored for ventilation, haemodynamic status, temperature and fluid and electrolyte balance (Fig. 1). This included urinary catheterisation and monitoring of bleeding and pleural effusion volumes. Data were recorded at 5-min intervals.

#### Experimental protocol

The s-ALI and ECMO procedures were performed as previously described [30, 37]. In brief, smoke produced by cotton in contact with a 750 °C metal plate was delivered with manual ventilation, 10–12 mL/kg tidal volume.

In preparation for ECMO, a 21–23-French femoral venous canula (Medtronic, Minneapolis, MN, USA) was percutaneously inserted and positioned in the distal inferior vena cava via the internal jugular vein (access canula). A 19–21-French venous cannula (Medtronic, Minneapolis, MN, USA) was percutaneously placed at the superior vena cava-right atrium junction (return cannula). Intra-cardiac echocardiography confirmed cannula position, method as published elsewhere [38].

ECMO was initiated two hours after s-ALI was induced (-6H). A Jostra ROTAFLOW centrifugal pump (Maquet Cardiopulmonary, Rastatt, Germany) was used, and target flow rates of 3–4 L/minute was maintained. Both the 3/8 PVC tubing and the Quadrox DTM oxygenator (Maquet, Germany) were BIOLINE coated. Porcine mucous heparin (1000 U; Pfizer Australia, West Ryde, Australia) was added to the circuit as a bolus with continuous infusion commenced at 4 U/kg/h. Fresh whole blood was collected in kaolin tubes to monitor activated clotting time using a HEMO-CHRON 401 coagulation analyser (Soma `Technology, Bloomfield, USA). A result between 220 and 300 s was targeted.

At 6 h post-ECMO initiation (0H), sheep in the two transfusion groups, but not the control group, were transfused. These sheep received 2 RBC units over 30 min via standard intravenous giving-sets with 200 μm filters (Alaris CareFusion BD, Hampshire, UK). At 18 h after transfusion initiation (18H), animals were sacrificed via pentobarbital.

Throughout the experiment, mean arterial pressure (MAP) was maintained > 65 mmHg, by protocolised use of pressors, inotropes and balanced fluid resuscitation. Bolus fluids and increased maintenance fluids (up to 500 mL/h) were administered when a persistent decrement in ECMO blood flow was observed, and other potential causes were excluded.

#### Sampling

##### Venous blood

Venous whole blood samples were collected at specified time-points (Fig. 1). Full blood counts (FBC) were performed using the veterinary mode of the AcT differential haematology analyser (Beckman Coulter Australia Pty Ltd, Lane Cove West, Australia).

Rotational thromboelastography (ROTEM) measurements were performed in accordance with manufacturer’s instructions (Werfen, Barcelona, Spain): EXTEM (thromboplastin-initiated coagulation), INTEM (contact factor-initiated coagulation), FIBTEM (thromboplastin-initiated coagulation with the platelet inhibitor cytochalasin D) and HEPTEM (contact factor-initiated coagulation with heparinase). To account for heparin administration, HEPTEM results were reported instead of INTEM. A ratio of HEPTEM to INTEM clotting time (CT) < 0.75 indicated a heparin effect was taking place. Animals that did not respond with a heparin effect were excluded from ROTEM analysis. Clot formation time (CFT, s) and maximum clot firmness (MCF, 10^–3^ m) were assessed over 30 min, at which point clot size (A30, 10^–3^ m) was evaluated. If clots did not form, values of 1800 (CFT) and 0 (MCF and A30) were assigned to facilitate statistical analyses. 1800 represents the maximum time (seconds) that samples were run.

Serum and platelet poor plasma were obtained (double centrifugation for 15 min, 4 °C, 3000 × g) and stored at − 80 °C for post hoc tests. IL-6, IL-8, and IL-1β plasma concentrations were quantified by in house ELISAs as described previously [39]. Samples were analysed in duplicate. Those below the detection limit were assigned a value of 0 for statistical analysis. Serum biochemical analyses were conducted with the Cobas C Integra (Roche Diagnostics, Basel, Switzerland) and its corresponding cassette (creatinine, total bilirubin, direct bilirubin, alanine aminotransferase and aspartate aminotransferase). Samples were tested in duplicate according to manufacturer’s instructions.

##### Lung pathology

Post-sacrifice, samples were taken from the lower lobes of both the left and right lung. Samples were assessed for wet-to-dry ratio, frozen at − 80 °C for RNA analysis or fixed in 10% buffered formalin for 24 h before paraffin embedment. For histopathological grading and polymorphonuclear leukocytes (PMN) counts, 5 µm sections were stained with hematoxlyin and eosin (H&E) using standard procedures. Initial histopathological analyses demonstrated that the right lung did not differ significantly from the left lung (data not shown). As such, all subsequent lung pathology was assessed for the left lung samples only.

*Histopathological Grading*. Under light microscopy, two independent investigators used a published protocol [40] to grade pathology for two samples (from the same lung lobe) per sheep. Pathology categories were pulmonary oedema, vascular and alveolar features, and bronchiole pathology. Grades were: 0 (normal), 1 (mild), 2 (moderate), and 3 (severe). Mild ratings had neutrophils in the blood vessels with mild alveolar oedema. Moderate ratings had additional features of extravascular inflammatory cell infiltrate and moderate alveolar oedema. Severe ratings had additional features of bronchiole damage with detachment of the lining and infiltration of inflammatory cells. Differences (> 1) between investigators were resolved by a third independent investigator. Scores from each investigator were averaged.

*Polymorphonuclear Neutrophil (PMN) and Macrophage Counts*. The total number of PMN and macrophages in 20 fields of view were assessed for two samples (from the same lung lobe) per sheep. An average of the samples was taken for reporting. PMN were identified by H&E stain under light microscopy. Macrophages were identified by immunohistochemistry as previously described [33].

*Gene Expression*. Total RNA was isolated using the RNeasy Mini Kit (Qiagen, VIC, Australia). Real-time reverse transcription polymerase chain reaction (RT-PCR) was performed in triplicate using primers for IL-6, IL-8, IL-10, IL-1β TNF-α, MMP2 and MMP9 (PrimerDesign, Southampton, UK). Normalisation protocol was as previously described [33].

##### Renal pathology

Kidney samples were processed for H&E staining as per Lung Pathology.

*Histopathological Grading*. Under light microscopy, one investigator examined for the presence of necrosis, luminal cast formation and epithelial calcification deposits. Severity was graded on whole number scale between 0 and 2.

### Statistical analysis

Unless stated, graphs present mean and standard deviation while tables and results text present mean with a 95% confidence interval. GraphPad Prism (Version 9.2.0) was used for statistical analyses. When comparing treatments alone, a one-way ANOVA with Bonferroni’s multiple comparisons test was used. When assessing treatments over a time course, two-way ANOVA was used if no data were missing whereas a mixed effects model was used if any data points were missing. Geiser-Greenhouse correction for sphericity and Bonferroni’s multiple comparison test were applied. Statistical significance was set as *p* < 0.05, using adjusted p values when applicable.

## Results

All time points are relative to the start of transfusion. A single sheep receiving fresh RBC transfusion was excluded before analysis due to a critical arrhythmia secondary to air embolism at 12H. This excluded experiment was terminated immediately at 12H and post-mortem analysis indicated potential previous thickening of the right and left ventricles. As indicated by ROTEM analysis, a single sheep in the control group did not respond to heparin. No discernible reason (e.g., coagulation factor deficiency) could be established. Data from this animal was excluded from ROTEM analyses only.

Mean sheep weights (kg) in the control group were 49.4 (46.2–52.7), compared to 45.4 (41.3–49.6) in the fresh RBC group and 52.5 (46.0–59.0) in the stored RBC group. Assessing RBC units pre-transfusion, significantly lower pH, sodium, and glucose, as well as significantly higher PO_2_, potassium and lactate were observed in the stored RBC units relative to the fresh RBC units (data not shown). This was as expected and due to the *ex-vivo* storage and metabolism of blood components, it represents one component of the storage lesion.

### No clinically significant differences in ventilation requirements

Tidal volume and compliance are measures of respiratory function that have been positively associated with survival in acute respiratory distress syndrome (ARDS) treated via ECMO [41]. In the present study, both tidal volume and compliance decreased, while positive end expiratory pressure (PEEP) and peak airway pressure increased (Table 1). Only peak airway pressure differed between treatment groups (*p* = 0.0025), with the stored RBC group experiencing lower pressures compared to the control group immediately prior to and after transfusion (0H: *p* = 0.0144, 1H: *p* = 0.0122).

**Table 1:** Ventilation Settings and Measurements

	ECMO	0H	1H	18H
Compliance (mL / cm H_2_O) !				
Control	31.59 (28.56–34.62)	18.25 (14.68–21.81)	16.34 (12.21–20.47)	7.31 (4.65–9.96)
Fresh RBC	31.62 (26.12–37.12)	19.44 (13.66–25.21)	14.7 (8.8–20.60)	7.7 (3.45–11.95)
Stored RBC	36.48 (31.01–41.95)	19.66 (14.36–24.95)	16.35 (11.47–21.23)	9.81 (6.29–13.33)
Tidal Volume (mL) !				
Control	469.99 (439.3–500.69)	286.44 (227.27–345.61)	261.07 (198.52–323.62)	168.45 (98.65–238.25)
Fresh RBC	467.8 (427.02–508.57)	265.37 (193.44–337.29)	208.9 (134.67–283.14)	148.88 (69.96–227.8)
Stored RBC	488.38 (442.79–533.97)	228.88 (155.62–302.13)	200.89 (132.99–268.79)	186.48 (119.28–253.67)
Max Pressure (cm / H_2_O) ! #				
Control	27.22 (25.71–28.72)	27.69 (24.86–30.52)	28.43 (26.27–30.59)	37.46 (32.16–42.75)
Fresh RBC	27.1 (23.87–30.33)	25.4 (22.18–28.62)	26.5 (24.1–28.91)	33.05 (26.2–39.89)
Stored RBC	24.97 (20.72–29.22)	22.73 (20.81–24.65) *	24.09 (22.01–26.16) *	30.38 (26.88–33.87)
PEEP (cm / H_2_O) !				
Control	10.01 (9.99–10.03)	10.62 (9.13–12.1)	10.57 (9.13–12.01)	12.75 (9.38–16.11)
Fresh RBC	9.29 (7.54–11.03)	10.00 (10.00–10.00)	9.95 (9.76–10.14)	11.43 (9.17–13.68)
Stored RBC	8.61 (6.73–10.5)	9.89 (9.74–10.03)	10.02 (9.96–10.08)	10 (9.96–10.04)

2-Way ANOVA with Bonferroni’s multiple comparison. If data sets were incomplete, mixed effects model with Bonferroni’s multiple comparison test

^a^ECMO represents ECMO pump initiation. ^b^0H is immediately prior to transfusion. ^c^1H is 30 min post-transfusion. ^d^18H is immediately preceding euthanasia. Data are recorded at 5-min intervals. Averages of the preceding 55 min are presented (95% confidence interval)

Max Pressure = peak airway pressure, PEEP = positive end-expiratory pressure

* = *p* < 0.05 vs. Control, ! = 2-Way ANOVA or mixed effects model time *p* < 0.05, # = Mixed effects model treatment *p* = 0.0025

### Pulmonary artery pressure increased after stored RBC transfusion

**Fig. 2:**
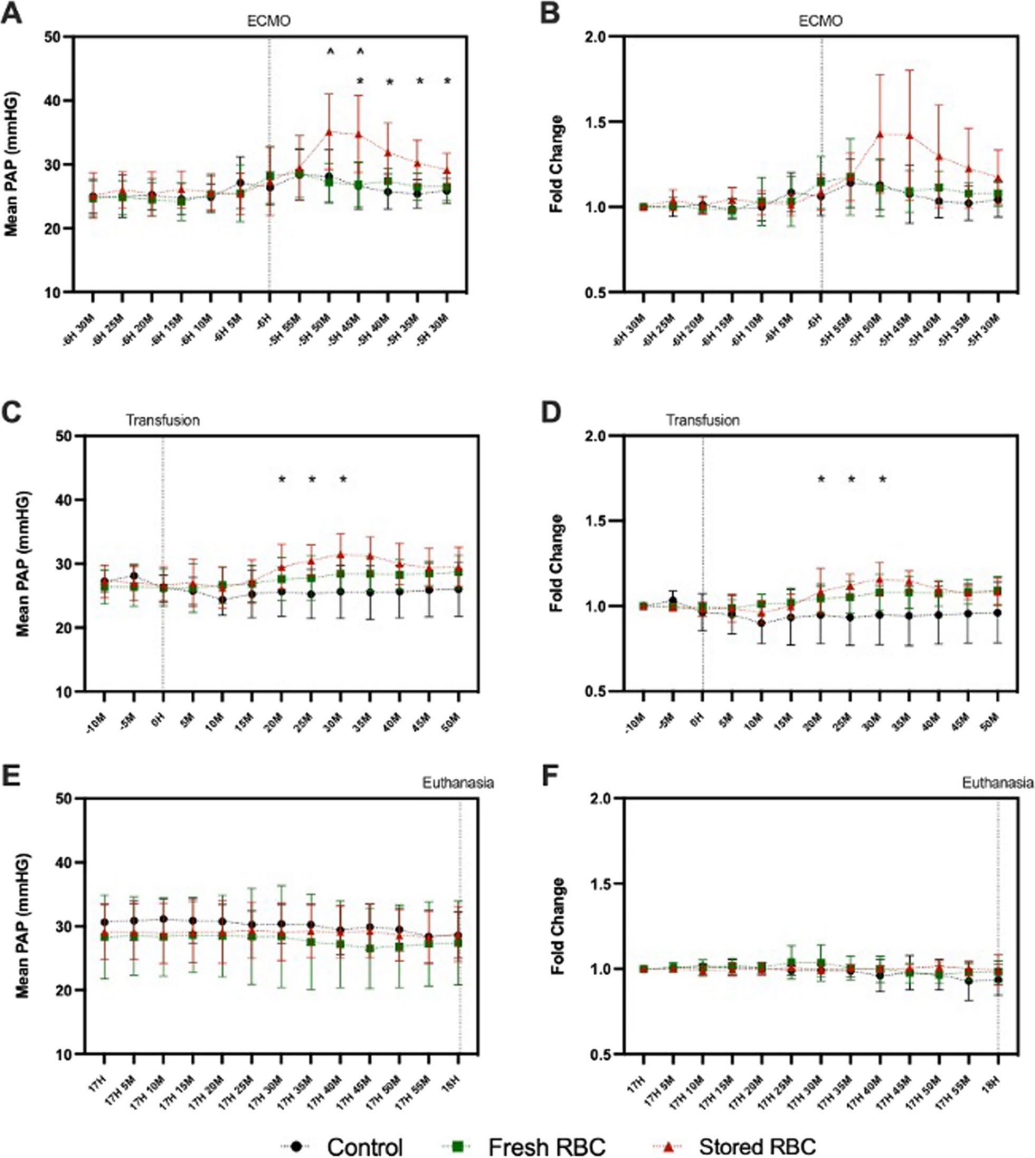
Mean Pulmonary Artery Pressure during ECMO Initiation, Transfusion, and Pre-Euthanasia. The initiation of ECMO (**A** and **B**) resulted in a large but highly variable increase in pulmonary artery pressure (PAP) in the stored RBC transfusion group (**A**). However, this was no longer evident when adjusted to fold change (**B**). After transfusion (**C** and **D**), an increase is again seen in the stored RBC group (**C**). When viewing fold change, this increase was less pronounced but more consistent than that which happened after ECMO initiation (**D**). PAP was stable for the hour preceding euthanasia (**E** and **F**). Control (black/circle, *n* = 8), fresh RBC (green/square, *n* = 7) and stored RBC (red/circle, *n* = 8). ECMO = extracorporeal membrane oxygenation and PAP = pulmonary artery pressure. Mixed effects model with Bonferroni’s multiple comparison. *p* < 0.05: * = Control vs Stored, ^ = Fresh vs Stored

Elevated pulmonary artery pressure (PAP) can be a result of ARDS [42] and stored RBC transfusion [43]. Transient increases in PAP were seen with ECMO initiation (-6H; Fig. 2A, B) and with RBC transfusion (0H–0.5H; Fig. 2C, D) but were no longer evident at the end of the experiment (17H–18H; Fig. 2E, F).

Immediately after ECMO initiation (-5H 45 M), mean PAP was higher in both transfused groups (stored RBC group (*p* = 0.0218) and fresh RBC group (*p* = 0.0299)) compared to the control group (Fig. 2A). While differences reduced over time, mean PAP in the stored RBC group continued to be significantly elevated compared to the control group until the last time point analysed for ECMO initiation (-5H 30 M; *p* = 0.0458). To control for these pre-treatment differences, we also investigated mean PAP expressed as a change relative to the -6H 30 M time-point and differences between groups were no longer apparent.

After initiation of the RBC transfusion, mean PAP was observed to be higher in the stored RBC group than the control group (Fig. 2C) at 25 M (*p* = 0.0204), 30 M (*p* = 0.0224) and 35 M (*p* = 0.0234). When mean PAP at these time-points was adjusted to change relative to the -10 M time-point, these differences between the stored RBC group and the control group were still apparent (*p* = 0.0442, *p* = 0.0376 and *p* = 0.0375 respectively). No differences in absolute mean PAP or change in mean PAP were observed between the fresh RBC group and either the control group or the stored RBC group.

### Stored RBC transfusion was associated with some evidence of lung injury

**Fig. 3:**
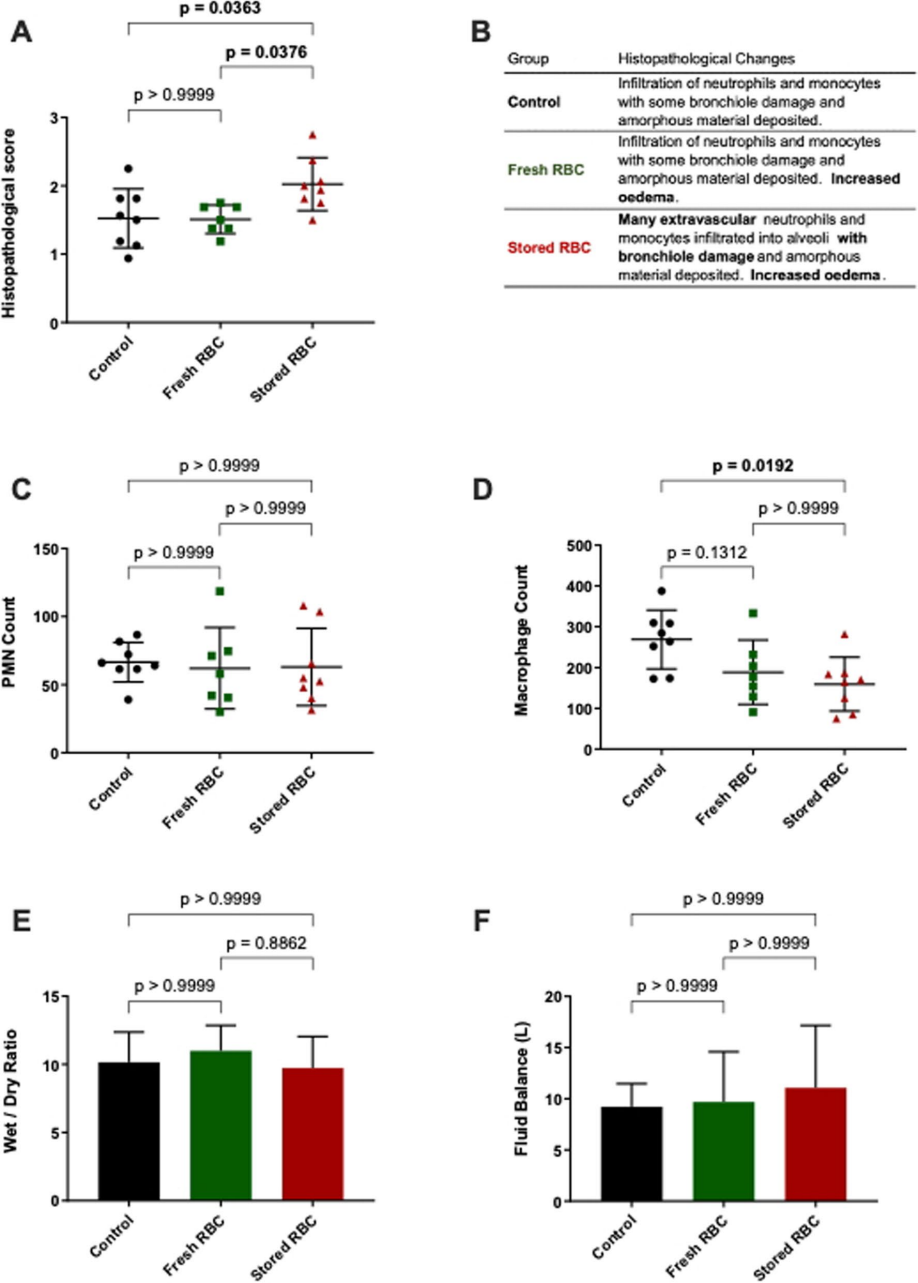
Post-Mortem Analysis of Histopathology; Injury, Cell Counts and Oedema (**A**) and summary descriptions (**B**) of histopathological grading showed increased bronchiole damage and extravascular neutrophil infiltration after stored RBC transfusion. Stored RBC transfusion was associated with no change in Lung parenchyma PMN (**C**), but decreased Macrophages (**D**) were observed. There were no differences present in Wet to Dry ratio (**E**) or fluid balance (**F**). Control (black/circle, *n* = 8), fresh RBC transfused (green/square, *n* = 7) and stored RBC transfused (red/circle, *n* = 8). PMN = polymorphonuclear leukocytes. One-Way ANOVA with Bonferroni’s multiple comparison. *p* values < 0.05 are bolded

As stored RBC transfusion has been implicated in the development of lung inflammation [44] and transfusion-related acute lung injury (TRALI) [45], we also assessed histopathological lung injury scores. These scores were higher in the stored RBC group (2.02, 1.7–2.35) compared to both the control (1.52, 1.16–1.89; *p* = 0.0363) and fresh RBC (1.51, 1.32–1.7; *p* = 0.0376) groups (Fig. 3A). This injury was characterised by greater extravasation of inflammatory cells as well bronchiole damage and oedema (Fig. 3B). However, there was no evidence of increased pulmonary oedema in the stored RBC group compared to the other two groups based upon lung wet-dry ratio. There were also no potentially causative fluid balance differences observed between groups. Counts of parenchymal PMNs and macrophages revealed lower numbers of macrophages in the stored RBC group when compared to the control group (*p* = 0.0192). No differences between groups were observed in the inflammatory responses measured within peripheral blood (ELISA: IL-6, IL-8 and IL-1β) and lung parenchyma (RT-PCR: IL-6, IL-8, IL-10, IL-1β TNF-α, MMP2 and MMP9) (data not shown).

### RBC transfusion was not associated with renal injury

**Fig. 4:**
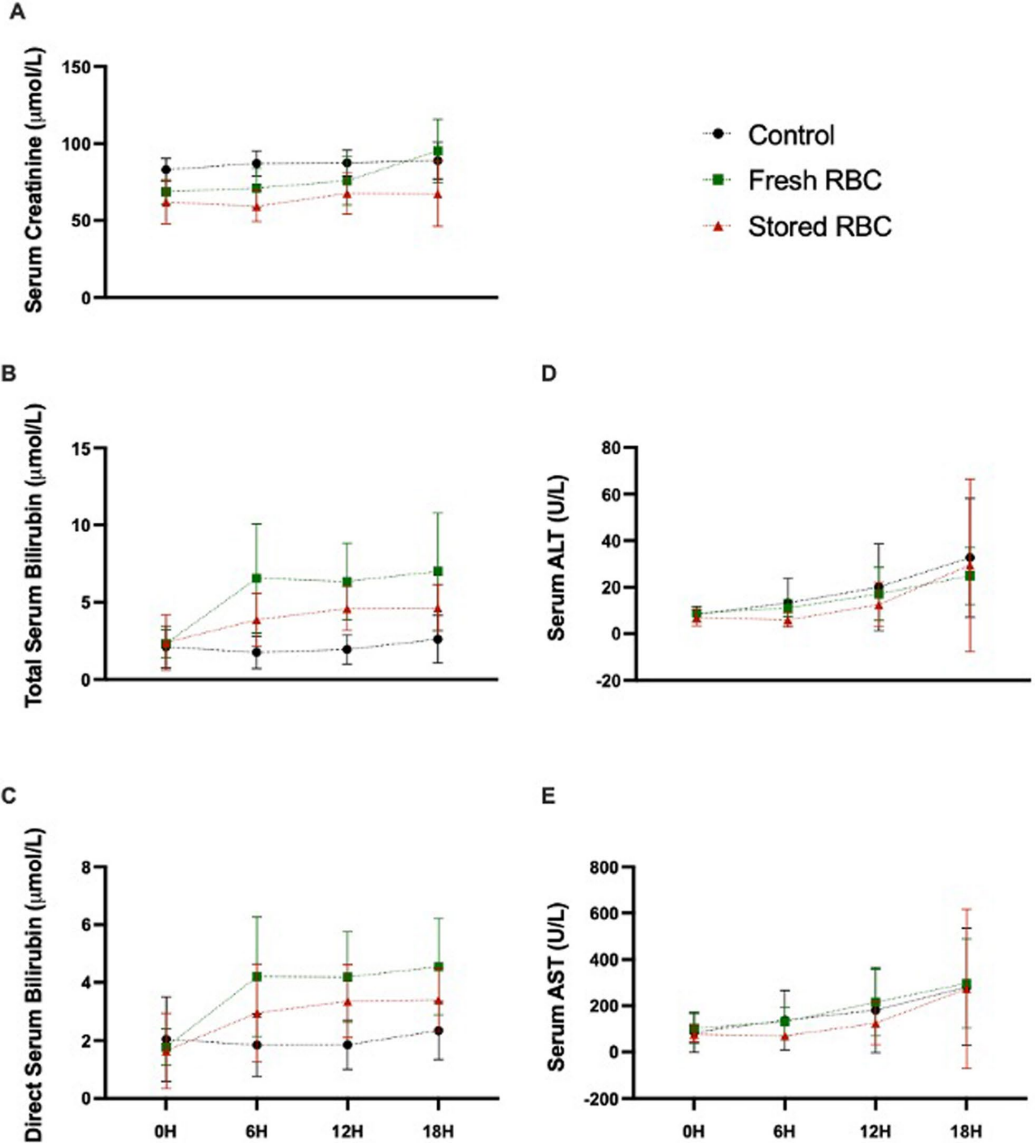
Serum Creatinine, Bilirubin, ALT, and AST. Serum creatinine levels (**A**) were initially different between transfusion groups and control. This difference was no longer observed for fresh RBC by 6H but persisted for stored RBC until 18H. Total serum bilirubin (**B**) was initially similar between groups, with an increase compared to the control group evident in the fresh RBC group at 12H and in the stored RBC group at 6H and 12H. Direct serum bilirubin (**C**) was initially similar between groups, with an increase compared to the control group evident in the fresh RBC and stored RBC groups at 12H. Alanine aminotransferase (ALT) (**D**) and aspartate aminotransferase (AST) (**E**) increased in a time dependent manner for all treatment groups but not treatment associated differences were present. Control (black/circle, *n* = 8), fresh RBC transfused (green/square, *n* = 7) and stored RBC transfused (red/circle, *n* = 8). Mixed effects models with Bonferroni’s multiple comparison. * *p* < 0.05 Control vs Stored: # *p* < 0.05 Control vs Fresh

Organ damage such as acute kidney injury can be a life-threatening risk of ECMO [46] with higher rates associated with RBC transfusion [47]. In our study, serum creatinine, which is an indicator of glomerular filtration, was lower in the fresh RBC (68.64, 61.39–75.89 µmol/L; *p* = 0.0131) and stored RBC (62, 50.27–73.73 µmol/L; *p* = 0.0110) groups compared to the control group (83, 76.08–89.92 µmol/L; Fig. 4A) immediately prior to transfusion (0H). At subsequent time-points (6H and 12H), the lower serum creatinine concentrations were still evident in the stored RBC group (*p* < 0.0001 and *p* = 0.013 respectively), but not in the fresh RBC group. By the end of the experiment (18H), serum creatinine was similar across the three treatment groups. Sections of kidney underwent histopathological assessment as a direct measure of renal injury; however, there was no evidence of treatment associated histopathological differences in necrosis, luminal cast formation or epithelial calcification (data not shown).

### RBC transfusion may be associated with hepatocellular injury

Alanine aminotransferase (ALT), aspartate aminotransferase (AST) and bilirubin levels are common clinical indicators of hepatocellular injury and/or dysfunction. Platelet transfusion has previously been correlated with elevated ALT and AST in the paediatric intensive care unit [48] and in our study, serum ALT and AST increased between pre-transfusion baseline (0H) and euthanasia (18H) in all three groups (Fig. 4D, E). Time associated differences were significant with *p* = 0.003 and *p* = 0.0059 for ALT and AST respectively. Treatment associated differences were not significant.

Total serum bilirubin levels (Fig. 4B) immediately prior to transfusion (0H) were similar amongst the three groups. Compared to the control group (6H: 1.75, 0.88–2.62 µmol/L; 12H: 1.94, 1.15–2.73 µmol/L), a transient increase was seen in the fresh RBC group at 12H (6.34, 4.05–8.63 µmol/L; *p* = 0.0076), and in the stored RBC group at 6 H (3.87, 2.43–5.31 µmol/L; *p* = 0.0357) and 12H (4.64, 3.42–5.76 µmol/L; *p* = 0.0022). Similarly, direct serum bilirubin levels (Fig. 4C) were similar between all three groups pre-transfusion, prior to a transient increase compared to the control group (1.85, 1.14–2.56 µmol/L) being observed at 12H in the fresh RBC (4.19, 2.75–5.64 µmol/L; *p* = 0.0191) and stored RBC (3.36, 2.3–4.41 µmol/L; *p* = 0.0469) groups**.**

### Coagulation disturbances appear transfusion storage independent

While intended to manage bleeding, transfusion has the potential to exacerbate coagulopathies [49] that underlie life-threatening haemorrhage in ECMO [3]. In the present study, coagulopathy was assessed using ROTEM (Table 2). EXTEM examines the extrinsic pathway of coagulation, and compared to the control group, the fresh RBC group had lower A30 at 6H (*p* = 0.0397), 12H (*p* = 0.0166), and 18H (*p* = 0.0108), as well as firmer MCF at 12H (*p* = 0.0306) and 18H (*p* = 0.0056). Similarly, compared to the control group, the stored RBC group had higher A30 at 12H (*p* = 0.0043) and 18H (*p* = 0.0122), and firmer MCF at 6H (*p* = 0.0309), 12H (*p* = 0.0061), and 18H (*p* = 0.0116). Additionally, the stored RBC group had shortened CFT at 12H (*p* = 0.0338) and 18H (*p* = 0.0469) compared to the control group.

**Table 2:** Rotational Thromboelastometry Evaluation of Intrinsic and Extrinsic Haemostasis

		0H	0.5H	1H	6H	12H	18H
EXTEM	A30 (10^−3^ m) #						
	Control	63 (58.8–67.2)	63.14 (59.75–66.54)	60.86 (55.56–66.15)	60.43 (57.02–63.84)	57.14 (54.1–60.18)	59 (55.54–62.46)
	Fresh RBC	66.29 (61.76–70.81)	64.43 (60.1–68.76)	64.43 (59.75–69.11)	66.14 (62.75–69.54) *	64.14 (60.17–68.12) *	66.29 (62.76–69.82) *
	Stored RBC	64.38 (61.93–66.82)	62.5 (59.97–65.03)	63 (59.54–66.46)	65.13 (62.46–67.79)	66.25 (61.92–70.58) *	67.38 (62.84–71.91) *
	CFT (S)						
	Control	199.29 (157.08–241.49)	178.43 (139.99–216.87)	193.29 (151.3–235.27)	211.43 (168.5–254.36)	226.29 (186.67–265.91)	213.71 (182.9–244.53)
	Fresh RBC	175.71 (141.07–210.36)	179.57 (136.24–222.9)	176.71 (130.71–222.72)	168.43 (138.65–198.21)	184.43 (131.82–237.04)	167.14 (118.68–215.61)
	Stored RBC	180.63 (157.8–203.45)	189.88 (159.26–220.49)	169.38 (144.48–194.27)	182.88 (161.21–204.54)	164 (132.98–195.02) *	155 (115.27–194.73) *
	MCF (10^−3^ m) #						
	Control	64.43 (60.16–68.7)	64.43 (61.56–67.3)	64.14 (58.93–69.36)	62.57 (59.24–65.9)	59.86 (57.01–62.7)	60.86 (57.77–63.94)
	Fresh RBC	67.43 (63.06–71.79)	65.86 (61.92–69.8)	67.14 (62.18–72.11)	67.43 (63.73–71.12)	66.14 (62.1–70.19) *	68.29 (64.92–71.65) *
	Stored RBC	65.25 (62.81–67.69)	63.75 (61.35–66.15)	66.25 (63.5–69)	67.88 (65.29–70.46) *	68.38 (64.09–72.66) *	69.5 (64.69–74.31) *
HEPTEM	A30 (10^−3^ m)						
	Control	61.71 (57.55–65.87)	61.14 (57.06–65.22)	61.14 (56.96–65.33)	58.86 (54.81–62.9)	58 (55.17–60.83)	57.86 (54.77–60.94)
	Fresh RBC	65.43 (60.28–70.57)	53.43 (30.81–76.05)	63.43 (57.49–69.37)	64.29 (60.13–68.45)	61 (55.99–66.01)	63 (58.41–67.59)
	Stored RBC	64.38 (61.59–67.16)	53.75 (35.36–72.14)	60.75 (57.66–63.84)	64.5 (61.86–67.14)	65.13 (60.95–69.3) *	66.75 (63.27–70.23) *
	CFT (S) #						
	Control	122.43 (95.87–148.99)	120.71 (94.73–146.7)	120.57 (90.78–150.36)	136 (99.6–172.4)	135.86 (117.3–154.41)	131.86 (108.02–155.7)
	Fresh RBC	98.71 (83.03–114.4)	106 (86.02–125.98)	107.71 (83.72–131.71)	101.14 (86.3–115.98)	111.86 (71.1–152.61)	97.71 (74.87–120.55)
	Stored RBC	104.5 (91.53–117.47)	113.5 (97.32–129.68)	108.25 (95.84–120.66)	105.63 (88.64–122.61)	91.63 (73.72–109.53) *	83.25 (66.31–100.19) *
	MCF (10^−3^ m)						
	Control	62.14 (57.76–66.53)	61.57 (57.24–65.9)	64.57 (61.46–67.68)	60.14 (56.31–63.97)	59.86 (57.16–62.55)	59.29 (56.8–61.77)
	Fresh RBC	65.71 (60.44–70.99)	63.14 (57.58–68.7)	66.14 (60.53–71.75)	65.57 (61.21–69.94)	62.71 (57.57–67.86)	64.14 (59.3–68.99)
	Stored RBC	64.5 (61.54–67.46)	62.38 (59.55–65.2)	63.75 (59.96–67.54)	67.13 (64.46–69.79) *	67 (62.55–71.45) *	68.25 (64.71–71.79) *
FIBTEM	A30 (10^−3^ m) !						
	Control	14.71 (12.6–16.83)	14 (11.8–16.2)	14.71 (12.6–16.83)	15.57 (12.91–18.23)	15.43 (12.61–18.25)	17.86 (16.13–19.58)
	Fresh RBC	17.71 (14.27–21.16)	16.29 (13.69–18.89)	15.57 (12.46–18.68)	18.57 (15.86–21.29)	20.57 (16.21–24.94)	23 (19.71–26.29) *
	Stored RBC	14.13 (11.78–16.47)	13.63 (11.63–15.62)	13.75 (11.92–15.58)	16.5 (13.78–19.22)	20.75 (18–23.5) *	23 (18.15–27.85)
	CFT (S) !						
	Control	1800 (1800–1800)	1800 (1800–1800)	1800 (1800–1800)	1800 (1800–1800)	1765 (1681–1850)	1800 (1800–1800)
	Fresh RBC	1,485 (975–1,995)	1,662 (1,324–2,000)	1,727 (1,547–1,906)	1,480 (1,085–1,875)	1,177 (458–1,896)	962 (274–1,650)
	Stored RBC	1,800 (1,800–1,800)	1,800 (1,800–1,800)	1,800 (1,800–1,800)	1,800 (1,800–1,800)	1,305 (794–1,816)	911 (367–1,455) *
	MCF (10^−3^ m) !						
	Control	14.71 (12.6–16.83)	14 (11.8–16.2)	14.86 (12.83–16.89)	15.71 (13.01–18.42)	15.86 (12.77–18.94)	18.14 (16.34–19.95)
	Fresh RBC	17.57 (14.12–21.03)	16.29 (13.53–19.05)	16 (12.62–19.38)	18.71 (16.11–21.31)	20.86 (16.64–25.08)	23.57 (20.12–27.03) *
	Stored RBC	14.25 (12.07–16.43)	13.88 (11.96–15.79)	13.5 (11.55–15.45)	16.75 (14.15–19.35)	21 (17.9–24.1) *	23.5 (18.23–28.77)

^a^EXTEM examines the extrinsic pathway of coagulation. ^b^HEPTEM examines the intrinsic pathway of coagulation and uses heparinase to account for the effects of heparin administered to the subject. ^c^FIBTEM studies the role of fibrinogen in isolation from platelets. ^d^If a clot did not form within 30 min, CFT was allocated a value of 1800s. Data are presented as mean (95% confidence interval). ^e^A30 = clot size (mm) at 30 min post-initiation. ^f^CFT = clot formation time which is the time until a clot with an amplitude of 20 mm is formed. ^g^MCF = maximum clot firmness (mm)

2-Way ANOVA with Bonferroni’s multiple comparison

* = *p* < 0.05 vs. Control, ! = 2-Way ANOVA time *p* < 0.05, # = 2-Way ANOVA treatment *p* < 0.05

HEPTEM examines the intrinsic pathway of coagulation while accounting for the impact of concurrent heparin administration. Compared to the control group, the stored RBC group had higher A30 at 12H (*p* = 0.0170) and 18H (*p* = 0.0016), and firmer MCF at 6H (*p* = 0.0116), 12H (*p* = 0.0218) and 18H (*p* = 0.001). Additionally, the stored RBC group had shortened CFT at 12H (*p* = 0.0036) and 18H (*p* = 0.0057) compared to the control group.

FIBTEM examines fibrinogen function, and compared to the control group, the fresh RBC group had higher A30 (*p* = 0.0239) and firmer MCF (*p* = 0.0231) at 18H. Compared to the control group, at 12H the stored RBC group had higher A30 (*p* = 0.019) and firmer MCF (*p* = 0.0428). There were no differences in fibrinogen levels between treatment groups and values were at the lower end of normal (data not shown).

These haemostatic differences between groups could be associated with haemodilution; however, no differences were found in the volume of intravenous fluids administered (data not shown), overall fluid balance (Fig. 2F) or haematocrit (data not shown). There were no indications of subsequent haemorrhage, as monitored by mean corpuscular volume (data not shown).

## Discussion

Patients receiving ECMO treatment are often transfused with many units of blood components, including RBC [12]. Refrigerated storage of RBC for up to 42 days is associated with development of a storage lesion that may impact the clinical outcomes in transfused patients [14]. Therefore, we aimed to investigate how the storage duration of transfused RBC impacted the outcomes of sheep undergoing VV ECMO after s-ALI. The key findings show that stored RBC was associated with significantly elevated pulmonary artery pressure and lung injury.

Chronic pulmonary artery hypertension has been associated with increased mortality in paediatric ECMO patients [50]. One of our principal findings was of a transient increase in PAP soon after stored RBC transfusion. Similar increases in PAP following stored RBC transfusion have been reported in animal models of obesity [23], non-traumatic haemorrhage [51], cardiac surgery [52] and haemorrhagic shock [53]. Increased PAP can be associated with imbalances in fluid load [54] [55]; however, we observed no differences in fluid loads between the groups of sheep. We speculate that it is possible that the increased PAP may relate to insufficient nitric oxide bioavailability (INOBA). Upon transfusion, increased levels of free haemoglobin present in stored RBCs scavenge nitric oxide resulting in pulmonary vasoconstriction and an increase in PAP [43]. Accordingly, inhaled nitric oxide administration ameliorated the effect of stored RBC transfusion upon increased PAP in previous studies [22, 43, 53, 56]. Nitric oxide is endothelial-derived, and the increased PAP observed following stored RBC transfusion was more severe in settings of endothelial dysfunction—obese humans [23] and a diabetic mouse model [56]. An ICU based study found endothelium dependent vasodilation to be a strong predictor of mortality [57]. Further studies, replicating the stored RBC arm of our study with and without inhaled nitric oxide treatment are needed to define the role of INOBA in the transfusion-associated increased PAP that we observed. We noted that the stored RBC group also had a higher PAP immediately following ECMO initiation; however, unlike the post-transfusion increase, once corrected for pre-event values, this was no longer evident. A highly variable PAP response following ECMO initiation has been reported previously [58], and may relate to the return cannula placement in the right atrium. Cannula flow rates for treatment groups were no different when relative to cardiac output but absolute flow rate values did differ.

More severe histological lung injury was seen after stored RBC transfusion compared to fresh RBC transfusion and to non-transfused controls. Stored RBC transfusion has been shown to induce transfusion related acute lung injury (TRALI) via a two-hit mechanism with cardiopulmonary bypass [59], cardiac surgery [44] and lipopolysaccharide priming in both sheep [40] and rats [60]. In a previous study, sheep undergoing s-ALI and ECMO treatment were observed to have increased pulmonary oedema (lung wet-dry weights) compared to ECMO-only controls and decreased pulmonary compliance compared to ventilation only controls; however, histological lung injury scores were not different between these groups [33]. Max airway pressure significantly increased with time across all groups, despite down-trending tidal volumes. From pre-ECMO to euthanasia, mean tidal volume values decreased 1.80-fold in controls, 2.24-fold in fresh RBC and 2.43-fold in stored RBC. This was likely counter-balanced by the compliance mean value decreasing to even greater levels. 4.32-fold in controls, 4.11-fold in fresh RBC and 3.72-fold in stored RBC. So, the question remains as to why we observed increased histological lung injury with RBC transfusion but not increased pulmonary oedema or decreased pulmonary compliance. As the wet-dry weight ratios we observed here were as high as those reported previously [33], it may be that all three groups could be considered to have severe pulmonary oedema. Similarly, all three groups of sheep here finished with average pulmonary compliances of less than 10 mL/cmH_2_O, similar in severity to those previously reported [33]. Thus, any changes related to stored RBC transfusion may not be apparent over the background of increased pulmonary oedema and decreased pulmonary compliance caused by the underlying s-ALI and ECMO support. A previous study of stored RBC transfusion in haemorrhagic shock showed reduced lung injury via the use of inhaled nitric oxide [53]. As discussed earlier, further studies that replicate our stored RBC transfusion arm with and without nitric oxide would help ascertain whether the lung injury we observed was related to the increased PAP observed in this group of sheep.

During ECMO, blood product transfusion has been associated with acute kidney injury [46]. Transfusion of stored RBC specifically has been associated with prolonged renal replacement therapy [61]. As RBC storage, transfusion and ECMO are all associated with haemolysis [23, 31, 47], cell free haemoglobin mediated renal toxicity [62] may be the underlying cause. We found no treatment associated differences in renal histopathology or serum creatinine. This contrasts with these previous studies comparing rates of acute kidney injury [46, 62]. The difference in results may be related to our post-transfusion monitoring period of 18 h. ECMO is typically an ongoing supportive treatment with ongoing transfusion. It is possible that clinically significant accumulation of cell free haemoglobin may be dose and/or time dependent.

Platelet transfusion has been associated with elevated ALT and AST [48], however, research regarding hepatic injury and RBC transfusion storage appears absent. While hepatotoxicity was related to transfusion, we found no relation to transfusion storage. Elevations in total and direct serum bilirubin occurred 6H post-transfusion with levels persisting until euthanasia (18H). RBC transfusion has been correlated with free bilirubin; however, it is unclear whether the ECMO circuit or s-ALI was inducing haemolysis thereby creating a transfusion need rather than a marker of transfusion induced injury [47]. The post-transfusion timing of bilirubin elevations in our study indicates that transfusion itself may be causative and as direct bilirubin was elevated, the cause is more likely due to hepatocellular dysfunction or cholestasis [63] rather than storage or transfusion associated haemolysis [23, 31]. During hepatocellular injury, we would expect associated changes in ALT and AST. While ALT and AST were elevated in a time dependent manner after transfusion, these levels were not significantly different to controls. As a result, we cannot be certain as to whether the differences seen in bilirubin levels are representative of an acute hepatic injury.

By the time of euthanasia, coagulopathies had developed in both transfusion groups relative to the controls, presenting as firmer clots and reduced time to clot. Looking at *in-vitro* and animal studies, stored RBC units are known to be pro-coagulant [64, 65]. A rat model of LPS associated TRALI found stored RBC transfusion was associated with coagulation and inhibited fibrinolysis in the systemic and pulmonary compartments [44]. These systemic and pulmonary coagulation disturbances were replicated in a cardiac surgery cohort experiencing TRALI [66]. While these results were storage time dependent [44], our study found no treatment associated differences. A result similar to a study investigating standard issue versus fresh (5 days or less) RBC [67]. In another sheep study, s-ALI and ECMO separately and synergistically had anticoagulant effects—producing a reduced clot firmness and increased time to clot [32]. Our results may in fact be reflecting the overwhelming and opposing impact of s-ALI and ECMO support. Clinical research investigating ECMO over days—not hours—has not found an association between ECMO duration and bleeding risk [68] and as such, the clinical significance of our findings are unclear. Where feasible, longer monitoring durations are needed.

As with any preclinical research, the present study had limitations that provide the basis for follow-up research in the future. The stored RBC group had statistically significantly lower peak airway pressure than the control group immediately preceding transfusion and in the 1 h afterwards. This has the potential to have contributed to the observed differences in pulmonary artery pressure and lung injury that we observed here. While our suggestion that insufficient nitric oxide bioavailability may be a contributing factor linking these two findings is reasonable given previous research [22, 43, 53, 56], further experiments, either using this model or in an alternative preclinical model, are required to test this hypothesis. This experimental model consisted of 24 h on ECMO, and for the transfusion groups, this included 18 h of post-transfusion monitoring. As we have acknowledged earlier, patients are typically on ECMO for much longer than 24 h [68]. However, acute transfusion reactions typically develop during or soon after transfusion, with TRALI being defined as acute lung injury that develops either during or within 6 h of transfusion [69]. Nonetheless, extending out the time-course of ECMO treatment and post-transfusion monitoring in a future study would provide further insight into the interaction between ECMO, RBC transfusion, and lung injury. It is also possible that ECMO was somewhat protective against stored RBC mediated lung injury as in its absence higher ventilatory parameters would have been required to maintain blood gas exchange. Inclusion of experimental groups of S-ALI + fresh or stored RBC transfusion in a follow-up study would allow this possibility to be investigated.

## Conclusion

In this sheep model of s-ALI, VV ECMO, and RBC transfusion, stored RBC transfusion was associated with increased PAP and lung injury. The transfusion-associated hepatic injury and coagulopathy observed were independent of RBC storage duration.

## Data Availability

The datasets used and/or analysed during the current study are available from the corresponding author on reasonable request.

## Abbreviations

A30: Clot size at 30-min
ALI: Acute lung injury
ANOVA: Analysis of variance
CBP: Cardiopulmonary bypass
CFT: Clot formation time
CT: Clotting time
ECMO: Extracorporeal membrane oxygenation
ELISA: Enzyme linked immunoassay
EXTEM: Thromboplastin-initiated coagulation
FBC: Full blood count
FIBTEM: Thromboplastin-initiated coagulation with the platelet inhibitor cytochalasin D
H&E: Haematoxylin and eosin
HEPTEM: Contact factor-initiated coagulation with heparinase
IL-1-β: Interleukin 1β
IL-10: Interleukin 10
IL-6: Interleukin 6
IL-8: Interleukin 8
INTEM: Contact factor-initiated coagulation
MCF: Maximum clot firmness
MMP2: Matrix metalloproteinase 2
MMP9: Matrix metalloproteinase 9
PAP: Pulmonary artery pressure
PEEP: Positive end-expiratory pressure
PMN: Polymorphonuclear leukocytes
RBC: Packed red blood cell
RNA: Ribonucleic acid
ROTEM: Rotational thromboelastography
RT-PCR: Real-time polymerase chain reaction
s-ALI: Smoke induced acute lung injury
SAGM: Saline adenine glucose mannitol
TNF-α: Tumour necrosis factor α
TRALI: Transfusion-related acute lung injury
VA: Veno-arterial
VV: Veno-venous
